# Multi-sensory information and simulator immersion: impact on decision-making performance, presence, and cognitive load

**DOI:** 10.1007/s00426-026-02305-6

**Published:** 2026-05-04

**Authors:** Zachariah G. Hoyne, Khaya Morris-Binelli, Sean Müller, Benjamin Piggott, Paola Chivers, Evan Dekker

**Affiliations:** 1 National School of Health Sciences, The University of Notre Dame Australia, Perth, WA Australia; 2 Centre for Smart Analytics, Federation University Australia, Ballarat, VIC Australia; 3 School of Education, The University of Notre Dame Australia, Perth, WA Australia; 4 School of Medical and Health Sciences, Edith Cowan University, Joondalup, WA Australia; 5 Academic Services and Support Directorate, Federation University Australia, Ballarat, VIC Australia

## Abstract

Immersive technologies such as 360-degree video virtual reality (360VR) provide unique opportunities to simulate representative environments to investigate decision-making in a safe and cost-effective manner. Inclusion of multi-sensory visual, contextual, and auditory information is important to enhance simulator psychological fidelity. Yet, the influence of increased sensory information on decision-making, presence, and cognitive load in 360VR compared to less immersive two-dimensional video simulators is unknown. This study investigated multi-sensory information, presence, and cognitive load across immersion conditions (360VR and two-dimensional video) in an exemplar sport-specific decision-making task. Fifteen higher-skilled and 15 lesser-skilled Australian Rules Football players completed a decision-making task which presented visual information only, visual and contextual information, and visual, contextual and auditory information. Overall, there were little significant performance difference between simulators. There was a significant decrease in decision-making performance across both skill levels and simulators as contextual and auditory information was added. Decision-making performance decline was more pronounced for lesser-skilled than higher-skilled participants, when they had to utilise contextual information to make riskier decisions to win the game. Significantly more accurate decisions were made when congruent, compared to incongruent, auditory information was presented, particularly in two-dimensional video. Perceptions of presence and cognitive load were significantly higher in 360VR than two-dimensional video, regardless of skill level, whilst across both simulators, cognitive load increased as sensory information was added. These findings indicate provision of multi-sensory information is more important for decision-making than simulator immersion and presence. Therefore, a focus on increasing simulator immersion should be done so with caution.

## Public significance statement

This study tested how visual, contextual, and auditory information affects decision-making in 360-degree video virtual reality (360VR) and two-dimensional video simulators. Whilst decision-making was not different in 360VR compared to two-dimensional video, it was rated by participants as more engaging and mentally demanding. When contextual information was added, less experienced players were more likely to make less accurate decisions. Furthermore, auditory information which was inaccurate lead to poorer decisions. These findings suggest that immersive tools like 360VR can be useful for assessing decision-making, but the information presented is key to evaluate, and potentially train, decision-making skill.

Decision-making is a crucial perceptual-cognitive skill required for expert performance in time pressured domains such as sport, law enforcement, and military operations (Ericsson et al., 2018). Decision-making involves selection of the correct response from an array of stimulus options (Williams and Jackson 2019b). In order to make these decisions, one must first anticipate or predict what will occur in the environment, from available perceptual information (Morris-Binelli and Müller 2017; Williams and Jackson 2019b). For example, in invasion sports like soccer, a midfield player must decide whether to dribble, short pass, or long kick the ball down field based upon the opposition team’s defensive positioning. Assessment and training of player decision-making in the field can be challenging for coaches, because in invasion sports, several players are needed to create patterns of play that are like competition scenarios, in order to equally challenge each player’s decision-making skill (Mangalam et al., 2023). Moreover, repeated exposure to patterns of play need to occur without increased risk of injury through physical overloading of players (Müller et al., 2023). To address these issues, two-dimensional (2D) video and extended reality (XR) simulators have become popular but their design is focused upon visual information (Faure et al., 2020), despite literature that indicates auditory, contextual, and visual information contributes to motor skill decisions made in spatial navigation (Shayman et al., 2025), language comprehension (Ye et al., 2025), and sport (Abernethy et al., 2001). Therefore, contribution of multi-sensory information to decision-making in sport requires further investigation, which will guide more comprehensive design of simulators to accelerate performance.

Traditionally, 2D video simulation has been used to understand the underpinning mechanisms of how experts make decisions due to the ease of capture and display of sports-specific scenarios. Typically, footage of game-like scenarios are filmed, edited to display developing patterns of play until a critical point at which a decision must be made (i.e., temporal occlusion), and presented via a projector screen (Morris-Binelli and Müller 2017; Williams and Jackson 2019b). Participants are required to watch each scenario and decide on the best option. For example, studies using 2D video simulation have reported higher-skilled performers are superior than their lesser-skilled counterparts at utilising the available visual information to anticipate opponent intentions and decide upon an effective course of action in soccer (Belling et al., 2015; Roca et al., 2011) and basketball (Gorman et al., 2013; Ryu et al., 2013) open field play. Furthermore, studies have reported that the need for superficial visual information such as contour, shape, and colour are not crucial for decision-making (e.g., Ryu et al., 2015). Rather, the capability to utilise relative player motion information, which researchers can simulate using point-light and blurred displays, is critical for expert decision-making (North et al., 2009, 2011; Ryu et al., 2015). Therefore, a key mechanism of expert decision-making is greater sensitivity to relative motion information (e.g., movement between teammates and opponents on the field of play) and the integration of this information with more complex memory structures developed due to extended experience within the domain (North et al., 2011; Williams et al., 2012). Accordingly, this facilitates the capability to predict what will occur in the developing pattern of play to guide more accurate decisions (Christensen et al. 2016; Williams and Jackson 2019b). Whilst these 2D video studies have made a useful contribution to the understanding of expert decision-making, they have focused upon use of visual information, but have not considered how other sensory sources, such as non-visual contextual and auditory information, contributes to decision-making.

There has been growing interest in how contextual information, which can be in the form of opponent action preferences, game context (e.g., score and time) and auditory cues, contribute to anticipation and decision-making skill. Again, 2D video simulation temporal occlusion has been used, where an opponent such as a cricket bowler or tennis player’s action is displayed until a certain time point and the participant predicts the outcome. Conditions are created where contextual information (e.g., field-placings or action tendencies) is presented that is either congruent or incongruent to the opponent’s kinematics. For example, studies have reported that experts have superior anticipation to lesser-skilled players when there is congruency between contextual and kinematic information such as pitch count in baseball (Paull & Glencross, 1997), court positioning in tennis return of serve (Loffing & Hagemann, 2014), positioning of players during field hockey goalkeeping (Morris-Binelli et al., 2021), field-placing in cricket batting (Runswick et al., 2019), and action tendencies to pass is soccer (Gredin et al., 2018). In addition, experts are still superior to lesser skilled individuals at using incongruent contextual and kinematic information, as anticipation tends to be impeded less in experts, than lesser-skilled players (Jackson et al., 2020; Runswick et al., 2019; Wang et al., 2019). These findings indicate the capability to integrate information sources (e.g., teammate and opponent relative motion with game context) and then utilise this information to inform decision-making is crucial for expert performance (Christensen et al., 2016).

Similarly, the availability of auditory information such as from the sound of racket-ball contact in tennis or teammate verbalisation of tactical information has been manipulated and displayed with 2D video temporal occlusion. For instance, studies have reported that kinematic information of actions presented with manipulation of the loudness of ball-racquet contact (Cañal-Bruland et al., 2018), opponent stepping sound in fencing (Allerdissen et al., 2017), and congruent auditory instructions to pass the ball in volleyball (Klatt & Smeeton, 2020), contributes to anticipation and decision-making skill. Further, Riches et al. (2021) reported higher-skilled lacrosse players can use only auditory information, in the form of verbal teammate instructions, to anticipate the actions of an opponent team more quickly than lesser-skilled players. Like contextual information in other forms, incongruent auditory instructions (Klatt & Smeeton, 2020) and physical exertion sounds such as grunting (Sinnett & Kingstone, 2010), have been reported to impede anticipation and decision-making, leading lesser-skilled participants susceptible to being deceived by the auditory information. Collectively, this line of inquiry has contributed to the proposition that athletes utilise Bayesian reliability-based strategies to facilitate use of different types of sensory information for decision-making (Gredin et al., 2023). A Bayesian perspective proposes contextual information (termed contextual priors) is continuously combined with incoming environmental sensory information in order to reduce the uncertainty of judgements (Körding, 2007). Experience in a domain (e.g., sport) facilitates sophisticated understanding of the reliability of information sources (e.g., contextual information and relative motion information) that are present during performance. As such, greater domain-specific knowledge provides experts with a superior capability to identify important information sources, assess its reliability and associated situational probabilities, and flexibly integrate this information for more accurate decision-making (Gredin et al., 2023). Similarly, the two-stage model of expert anticipation (Morris-Binelli & Müller, 2017; Müller & Abernethy, 2012) also emphasises that the capability to switch between contextual and kinematic information is crucial for expert anticipation and decision-making. Importantly, despite the fact that multi-sensory information (e.g., game score and time, auditory cues from teammates) is continuously available during in-situ motor skill performance, its contribution to decision-making has not been simulated simultaneously in 2D video simulation studies. Further, potential limitations of 2D video simulation is that it presents only two-dimensional visual information, does not completely immerse the user in the simulated environment, and does not allow rotation of the head that occurs in invasion sports to use available visual information (Faure et al., 2020). XR has become popular as it can accommodate these limitations of 2D video simulation, but whether these advantages are significantly beneficial to decision-making and anticipation has not been systematically investigated.

In the past decade, there has been a significant increase in the use of XR simulation to further understanding of decision-making and anticipation skill in sport (see Faure et al., 2020; Kittel et al., 2024). XR is an umbrella term used to describe photo-realistic and virtual environments which are created by computer technology (Le Noury et al., 2022). A popular XR technology that has been used is 360-degree video virtual reality (360VR), which presents a 360-degree video of the real world to the user in a head-mounted display (HMD). Another popular type of XR is animated virtual reality (AVR), which presents computer generated synthetic virtual environments to a user in a HMD (Le Noury et al., 2022). Benefits of 360VR compared to AVR is that it is more accessible and cost-effective as specialised software programming expertise is not required to create synthetic virtual environments and that it can present more visually realistic information to the user (Müller et al., 2023; Robertson et al., 2023). Additionally, it is suggested that immersion (physical masking from the real world), presence (subjective perception of being transported to a different environment), and increased physical fidelity (realism of the visual display) created in XR, may influence decision-making performance in sport, as these technological features can more accurately represent the perceptual and motor components of real-world performance situations than 2D video (Düking et al., 2018; Kittel et al., 2024). This is important as according to representative task design (Krause et al., 2019), simulated environments with greater representativeness are proposed to elicit behavioural responses that are closer to those observed in the real-world performance environment and have a greater likelihood of facilitating positive transfer of learning from training to competition environments (Le Noury et al., 2022). It is important to note that although 360VR does not allow representative action responses, the increased immersion, presence, and presentation of visually realistic information likely increases the representativeness of the perceptual information, which is crucial for expert decision-making, compared to 2D video simulators (Müller et al., 2023). Relatedly, recent qualitative research has indicated some athletes prefer XR (i.e., 360VR and AVR) to 2D video simulation as a tool to train perceptual-cognitive skills, such as decision-making (Kittel et al., 2026). However, in regard to performance, Kittel et al. (2019) reported that expert umpires were superior in their decision-making to lesser-skilled umpires in 360VR and 2D video simulation tasks that presented visual information only. This suggests that decision-making performance is similar across these two simulation modalities, but direct comparison of performance between the two modalities was not conducted. Alternatively, task engagement was superior in 360VR compared to 2D video simulation. Therefore, it appears that presence and immersion, which are crucial technological features related to psychological constructs of XR, are independent factors to performance, but this may vary as multi-sensory information is simultaneously simulated (i.e. high psychological fidelity; Harris et al., 2020). Again, this could be due to the greater representativeness of the perceptual information presented in 360VR compared to 2D video simulation.

Inclusion of multi-sensory information into XR simulators has been scarce (Kittel et al., 2024), but may provide better insight into the interplay between presence, immersion, and performance. Two studies have included more than visual information in XR tasks. In AVR, Helm et al. (2020) manipulated probabilities of contextual and kinematic information in a handball defensive task and reported novices switched to use the most reliable information for anticipation. Like 2D video simulation tasks, Magnaguagno et al. (2022), using an interactive Cave Automatic Virtual Environment (CAVE), reported that experts were superior to lesser-skilled players at using congruent contextual and kinematic information in a handball defensive decision-making task. In both studies, presence was not measured, and immersion was not manipulated, so it is not possible to determine their impact upon the interplay between sensory information use and presence for performance. Overall, multi-sensory information including visual, contextual, and auditory are important for expert performance, but given they have not been simultaneously modelled and manipulated in XR, limited understanding of such sensory information use relative to immersion, presence, and performance is available.

Inclusion of multi-sensory information into XR simulation that increases immersion, presence, and representativeness of sport environments, could increase user cognitive load that may affect decision-making performance (Champion et al., 2023). Cognitive load refers to the contribution of a task on working memory, with load comprised of intrinsic (task complexity), extraneous (sensory information sources), and germane (cognition) components (Paas et al., 2003; Sweller, 2010). As per Cognitive Load Theory, it is proposed that an individual’s memory capacity is finite, and may become overwhelmed by increased extraneous and germane loads (Paas et al., 2003; Sweller, 2010). It has been argued that experienced performers are more resilient to decreases in performance as a result of increases in extraneous cognitive load as they can automatically integrate task-relevant information with existing schemata to maintain performance (Kalyuga et al., 2003). Although some 2D video tasks, which investigate cognitive load in a uni-sensory soccer interception task (Gredin et al., 2020), and with the provision of bi-sensory visual and contextual information in a cricket simulator (Runswick et al., 2018) have shown limited expertise differences, it is believed that the immersive nature of XR is likely to increase cognitive load (Ochs & Sonderegger, 2022), which is likely to be increased further with the inclusion of multi-sensory information. Recent research has investigated the impact of immersion on cognitive load and decision-making performance in a uni-sensory 360VR simulator to a task and sensory-matched 2D video task (Hoyne et al., 2026) within a single skilled cohort, however, the impact of expertise and multi-sensory information on decision-making performance and cognitive load across high and low immersion is still unknown. Therefore, as simulator design progresses to multi-sensory information it is crucial to consider how the influence of cognitive load affects performance.

The purpose of this study was to investigate the influence of immersion (360VR and 2D video), addition and congruency of multi-sensory information, and expertise on decision-making performance, perceptions of presence, and cognitive load. Australian Rules Football (ARF) was used as the exemplar invasion sport, where participants were required to watch representative play sequences and decide upon the optimal passing option to a teammate. ARF involves two teams of eighteen players that compete against each other on an oval playing field. The objective is to transfer the ball from one position on the ground to another using a handpass or kick and then kick the ball through upright posts to score more points than the opposition. Given published evidence, the following five hypotheses were formulated. First, it was predicted that expertise would influence multi-sensory information use (vision only to vision-contextual to vision-contextual-auditory) for decision-making as experts are better able to integrate non-visual sources of information with visual cues to guide anticipation and decision-making (Morris-Binelli et al. 2021; Runswick et al. 2018; Williams and Jackson 2019a), with performance higher in 360VR compared to 2D video due to greater immersion (Ochs & Sonderegger, 2022). Second, it was predicted that expertise would influence contextual information use for decision-making due to greater schema development and understanding of the competitive context (Kalyuga et al., 2010; Magnaguagno et al., 2022), with performance higher in 360VR compared to 2D video due to the underlying immersive properties of the display (Ochs & Sonderegger, 2022). Third, it was predicted that expertise would influence auditory information use, as skilled performers can better integrate auditory information with previously learned schema and current visual information to limit the susceptibility for being deceived by incongruent auditory information (Klatt & Smeeton, 2020), with the underlying immersive properties of 360VR to make this difference more pronounced compared to 2D video (Ochs & Sonderegger, 2022). Fourth, it was predicted that increasing multi-sensory information (vision only to vision-contextual to vision-contextual-auditory) would, in turn, result in an increased perception of presence in 360VR compared to 2D video simulation due to the interplay of immersion and task-specific information creating a more realistic and life-like representation of competition (Harris et al., 2020; Ochs & Sonderegger, 2022), with no influence of expertise. Fifth, it was hypothesised that cognitive load will be higher in lesser skilled compared to higher skilled players across multi-sensory information as they are less likely to integrate more stimuli, and process information in isolation, thereby incurring higher extraneous load in tasks (Paas et al., 2010; Sweller, 2010), with increased cognitive load in 360VR compared to 2D video (Ochs & Sonderegger, 2022).

## Method

### Participants

Thirty male ARF athletes from a single semi-professional state-level ARF club were recruited for this study. This cohort consisted of 15 higher-skilled (*M*_age_ = 21.87, *SD* = 2.93, *M*_years played_ = 13.33, *SD* = 5.25) males from the 1st and 2nd division adult men’s teams, and 15 lesser-skilled (*M*_age_ = 17.33, *SD* = 0.95, *M*_years played_ = 9.87, *SD* = 3.04) young adult males from the club’s development team. Participants provided informed consent, detailing their colour vision status, vision correction, vertigo or balance disorders, and recent concussions. Exclusions were made for individuals who were colourblind, had uncorrected vision, or had experienced a concussion within the past 12 days, in line with International Conference on Concussion and AFL protocols (McCrory et al., 2017). These exclusions aimed to reduce the impact of concussion-related cognitive impairment and perceptual information challenges on task performance.

An a priori power analysis was conducted in G*Power (Version 3.1.9.7) for the comparison of decision-making performance in 360VR and 2D video. This analysis indicated for a repeated measures analysis with α = 0.05, 80% power, and 95% confidence interval, a minimum of 15 participants in each skill group with 72 trials per task modality (144 total trials per participant) were required to detect a small effect (*f =* 0.14). For the comparison of decision-making performance in the vision-contextual and vision-contextual-auditory conditions, 15 participants in each skill group with 24 trials per condition for each modality could detect a small-to-moderate effect size (*f* = 0.21). Additionally, a priori power analyses for the comparison of presence and cognitive load across 360VR and 2D video tests indicated that with a sample size of 15 participants in each skill group, a small-to-moderate effect size (*f* = 0.20) could be detected. Ethics approval for this study was obtained from the lead author’s institutional ethics committee (Reference number: 2022–140F). Written informed consent was obtained from the participants and guardians of minors (aged under 18 years) prior to participation.

### Creation of the decision-making task

In collaboration with three expert ARF coaches (Level 3 Accredited) from a single semi-professional club, the researchers created and filmed an ARF decision-making task (see Hoyne et al., 2026 for details). In ARF, kicking the ball to a teammate who then catches (marks) the ball before it lands on the ground is one main attacking strategy. Once a player has marked the ball, opposition players are unable to encroach into a protected area around the individual with the ball (which extends 10 m either side of the player with the ball; Australian Football League, 2025). After the ball is marked and the umpire signals a mark has been taken, the player in possession of the ball has a limited amount of time (approx. 6–8 s; Appleby & Dawson, 2002) to identify a teammate to dispose the ball to, by a kick or hand, to move the ball down the field in order to score points. If this time has elapsed and the player in possession of the ball has not disposed the ball, the umpire calls “play on” and opposition players are free to tackle the player with the ball in order to dispossess them of it and gain possession of the ball for their team in order to try and score (Australian Football League, 2025). Accordingly, our task targeted the decision-making window of when a player marks the ball until the time when “play on” is called.

**Fig. 1:**
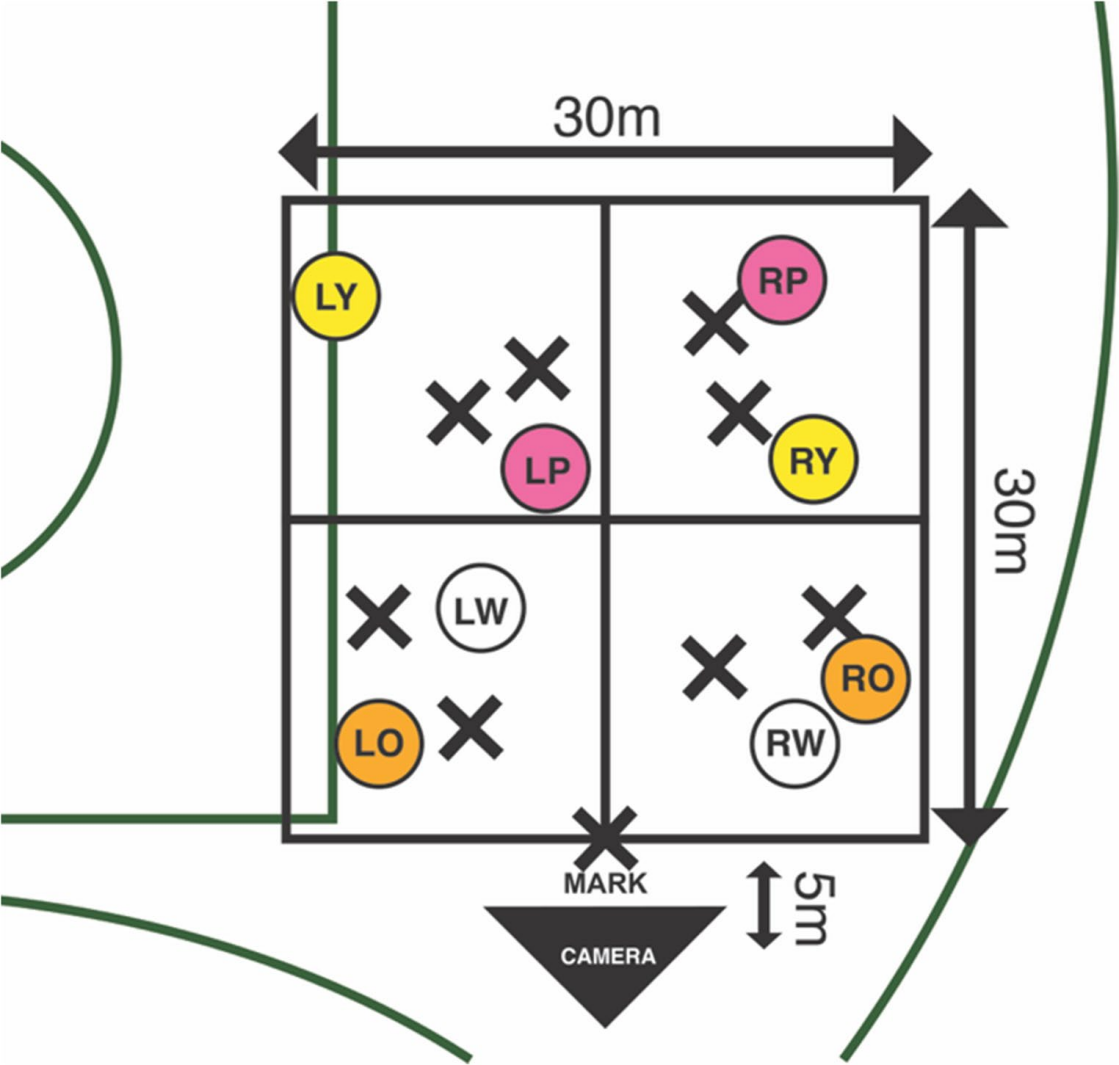
Schematic of the decision-making task on the field of play. X marks denote the defending players and circles denote attacking players. LW = left white, LO = left orange, RW = right white, RO = right orange, LP = left pink, RP = right pink, LY = left yellow, RY = right yellow. From Hoyne et al. (2026)

The decision-making test consisted of eight attacking players and eight defending players performing different running patterns in a 30 × 30 m zone that was located on the side of the centre square (located in the middle of the ARF playing field) between the two 50 m arches of an ARF field (see Fig. 1).

This playing area was further divided into four sub-zones, with each containing two attacking and two defending players. At one end of the playing zone, another defender stood on the mark (see Fig. 1). The defending players wore dark clothes to ensure a clear visual distinction between the attacking players who wore light coloured clothing underneath an orange, white, yellow or pink singlet. The coloured attacking actors ran pre-determined patterns calling for the ball as if the camera (participant) had possession of the ball. The aim of the test was for the participant to verbalise who to pass the ball to, hence there were 8 options available. Two players who took part in the filming to create the task stimulus were participants in the current study. However, during filming all players were blinded to the outcomes of each running pattern and filming of the decision-making task occurred approximately one year prior to the current study. Based upon previous research that has investigated decision-making in sport (Morris-Binelli et al., 2025; Piggott et al., 2019), the three coaches watched the entirety of each scenario over two sessions and were scored from the best decision (8 points) to the worst decision (1 point) until 100% agreement was reached for each clip.

#### Creating contextual and auditory stimuli

Contextual and auditory information used for this experiment was discussed and agreed upon by the research team and two new expert ARF coaches (Level 2 and 3 Accredited) from the same club involved in this broader research project. Regarding contextual information, both available game time and score were manipulated to change the context of the scenarios viewed in the decision-making task. In ARF, to win the game, a team must have the most points scored at the end of the game compared to the opposition. To do this, the ball must travel by a kick through the two large posts of the attacking goals to score 6 points or through small posts to score 1 point. At senior level, games consist of four quarters of 20 min playing time. Two contextual scenarios were designed, which were believed to change the decision-making of players, however, the running patterns did not change, but there were differences in the ranking of the best to worst options to select. In the *neutral* condition, participants were to select the player that had the greatest separation from their direct opponent. In the *aggressive* condition, participants were to change their decision-making to prioritise options further away from themselves (towards their attacking goals) and options in the middle of the ground to increase their team’s likelihood of scoring to win the game. The neutral and aggressive contextual conditions changed relative to the amount of time left in the final quarter of play, and whether the participant’s team was ahead or behind in the score. Therefore, in neutral conditions the participant’s team was ahead by more than four goals (24 points) at the beginning of the final quarter of play. In aggressive conditions, the participants team was losing by less than a goal (under 6 points), with less than 5 min remaining in the quarter. Changes in decision-making scoring relative to context changes was discussed with the two expert ARF coaches until 100% agreement was reached (see Supplementary Material A).

In relation to the auditory stimuli, congruent and incongruent audio of a teammate continuously instructing the participant which specific option to pass the ball to (e.g., “Left Yellow”) was created in collaboration with the two expert coaches, as this was deemed to be how players are instructed to communicate during competition. This simulated teammate would either provide the participant with congruent information (i.e., instructed to pass the ball to the best option) or incongruent information (i.e., instructed to pass to the worst option available) to the viewed play. This audio was recorded using an omnidirectional wireless microphone transmitting to a wireless receiver (RØDE GO II, RØDE, Sydney Australia).

A 360-degree stereoscopic video camera (Vuze 3D 360 4K VR Camera, Humaneyes Technologies Ltd., Neve Ilan Israel) sampling at 30 frames-per-second was used to obtain the video footage for the experiment. This camera was fastened to a tripod at a height of 1.7 m and 5 m from the intersection of the front playing zones. During filming, the attacking players were instructed to run and call for the ball as if the 360-degree camera was their teammate in possession of the ball, whilst the defensive players defended an attacking player. The resulting pool of 64 unique clips (8 running patterns × 8 unique versions) which were deemed adequate by the research team and the two coaches were used to create the test and familiarisation trials. However, due to the contextual stimuli, two of the running patterns were removed from the pool as it was deemed that there was no change in decision-making from neutral to aggressive conditions. Therefore, 30 new variations of the running patterns were selected from the pool of 64. Of these 30 running patterns, 24 were included in the test proper and 6 were used as familiarisation and practice trials. All running patterns included in this study were different to Hoyne et al. (2026).

#### Creation of 360VR and 2D video tests

Video files of the filmed test stimuli were transferred from the 360-degree video camera to a laptop computer (MacBookPro 18,3, Apple Inc., California United States of America). These videos were processed using Vuze VRStudio (Humaneyes Technologies Ltd., Neve Ilan Israel) and were rendered as a 360-degree video (16:9 aspect ratio), comprising standard stitching, no stabilisation, H.264 codec, auto optimal bitrate, equirectangular over/under, and stereo audio. The videos were then transferred into Adobe Premiere Pro (Version 23.1.0, Adobe Inc., California United States of America) to create the ARF decision-making video test. The test matrix consisted of 6 (scenarios) × 4 (unique versions) × 3 (sensory conditions) = 72 trials for each of the 360VR and 2D video tasks, with the same unique versions of each scenario presented across task modality.

To investigate multi-sensory information use, three sensory conditions were created: (i) vision only (s1), (ii) vision-contextual (s2), and (iii) vision-contextual-auditory (s3). In s1, only visual information in the form of the scenarios unfolding without additional contextual or auditory information were presented. In s2 and s3, time of game contextual information was displayed via a countdown timer indicating the time remaining in the final quarter of the match. In addition, game score contextual information was presented as the number of points the attacking team were ahead or behind. The first 12 trials presented neutral contextual information, and the remaining 12 trials presented aggressive contextual information. In s3, congruent or incongruent auditory information was added via the provision of a verbal instruction indicating to the participant which option (teammate) to dispose the ball. The auditory information was presented in a randomised order, ensuring an even spread of congruent and incongruent audio for each of the neutral and aggressive contextual conditions. Furthermore, all irrelevant background audio captured during the filming remained in s3, but was removed from s1 and s2 trials.

Familiarisation trials were also created and consisted of participants viewing the 6 running patterns, as well as 4 vision-contextual conditions (2 × neutral and 2 × aggressive), and 4 vision-contextual-auditory conditions (one of each context and audio combination, e.g., neutral context with congruent audio) for a total of 14 familiarisation trials. In addition, four practice trials were shown including one trial as vision only, one aggressive vision-contextual, and two vision-contextual-auditory conditions. These trials enabled the participant to practice verbalising their response, with the correct response shown to the participant prior to the commencement of the next trial. The video footage displayed in the familiarisation and practice trials were different to the footage included in the test proper for both 360VR and 2D video.

In 360VR, the trial number was displayed in the front, left, and right of the viewing perspective of the participant for 1 s. The front number was displayed in yellow to aid participant orientation in the HMD. In contrast, the 2D video test only presented the yellow trial number centrally on the screen. Subsequently, the first frame of the scenario was shown for 1 s to aid participants to orient to each trial, followed by the entire scenario clip (~ 6–8 s). An intertrial interval (ITI) of 5 s was included between each trial, which consisted of an audio cue after four seconds to signify to participants the approach of the subsequent trial. For s2 and s3, contextual information was displayed for 3 s after the trial number and before the still image of the scenario (e.g., “4th QUARTER - TIME REMAINING 5:28 − 4 POINTS AHEAD”). To reduce order effects, each sensory condition block of 24 trials was counterbalanced using a Latin Squares approach for each modality. To minimise familiarisation with the test stimuli, the scenarios within each sensory condition were randomised. Therefore, three counterbalanced decision-making tests were created for both 360VR and 2D video, resulting in 6 total tests. After each sensory condition, the text “Begin Questionnaires” was displayed on a black screen to enable participants time to pause the video, remove their HMD (if applicable), and begin their written responses to the Independent Television Commission-Sense of Presence Inventory (ITC-SOPI; Lessiter et al., 2001) and Rating Scale for Mental Effort (RSME; Zijlstra, 1993).

#### Independent television commission-sense of presence inventory (ITC-SOPI)

The ITC-SOPI was used to measure presence in each sensory condition across 360VR and 2D video. This questionnaire consists of 38 questions each on a 5-point Likert Scale ranging from (1) strongly disagree to (5) strongly agree and measure four factors: (i) spatial presence, (ii) engagement, (iii) ecological validity, and (iv) negative effects (i.e., cybersickness). Spatial presence is the feeling of being *within* the simulated environment and indicates being transported into and surrounded by the simulated environment. Engagement refers to the degree to which the user feels involved with and enjoys the simulated environment. Ecological validity is a measure of how lifelike and real the simulated environment is perceived to be. Lastly, negative effects consists of dizziness, nausea, and eyestrain as a result of viewing the simulated environment (Lessiter et al., 2001). The ITC-SOPI is a reliable method of measuring presence in XR as each category has high Cronbach’s alpha scores (spatial presence = 0.94, engagement = 0.89, ecological validity = 0.76, negative effects = 0.77; Lessiter et al., 2001). For spatial presence, engagement, and ecological validity, higher scores (i.e., closer to 5 on the 5-point Likert scale) indicate greater levels of these factors, whilst for negative effects, lower scores (i.e., closer to 1 on the 5-point Likert scale) signify fewer negative effects.

#### Rating scale for mental effort (RSME)

The RSME was used to measure cognitive load in each sensory condition across 360VR and 2D video. The RSME is a one-dimensional linear scale ranging from 0 to 150. Zero indicates no effort at all, 75 indicates moderately effortful, and 150 indicates very effortful. This valid and reliable scale has been used in research measuring cognitive load across many domains, such as sport (e.g., Runswick et al., 2018).

### Procedure

The administration of the 360VR and 2D video tests followed the same procedure as Hoyne et al. (2026). That is, each participant individually completed the 360VR and 2D video tasks in a counterbalanced order. Further, the 360VR task was presented via a Meta Quest 2 (Meta, California United States of America) HMD and the 2D video task was projected onto a 150-inch screen displayed from a laptop computer using VLC Media Player (Version 3.0.18, VideoLAN Association). This program was used as it can play 360-degree video in a 2D format and is freely accessible. At the beginning of the 2D video test, a keyframe image was presented prior to the test beginning and was used to zoom out the video so that participants could view the entire playing area of the task. For the 2D video task, participants stood at a mathematically calculated distance of 2 m away from the wall to replicate the viewing angle (19°) in a match. In both the 2D video and 360VR tasks, a video camera (GoPro HERO9, GoPro Inc., California United States of America) with audio function recorded the participant’s verbal responses for later coding. Verbal responses are considered an acceptable method to discriminate expert decision-making performance as they can activate motor regions in the brain (Aglioti et al., 2008; Kalén et al., 2021). To help orient participants to the XR experience and using the controllers, participants were given the opportunity to use the pre-installed “First Steps” application on the HMD for 5 min before beginning the 360VR task. Thereafter, the participants completed the familiarisation and practice trials before the test proper, which took an additional 5 min.

In both the 360VR and 2D video, participants were informed that the aim of the decision-making task was to select the best option (from the 8 available) on each trial and that the speed at which the response was made did not affect the scoring. Speed of participants’ responses were not a factor in the current study as the expert coaches indicated faster decision-making in mark-disposal scenarios does not always result in superior performance. Further, participants were informed that during the 360VR and 2D video tasks they would be provided with game score and time information as well as a teammate who would verbally instruct them to pass the ball to a particular teammate. No other information was provided to participants regarding the purpose of the contextual information, nor were the participants informed that the audio heard from the virtual teammate would be congruent / incongruent. Accordingly, participants were free to respond at any stage during the trial, but were reminded to respond before the next trial began, even if they were completely uncertain (~ 13 s to make a response). On each trial, participants verbalised their decision of which teammate to pass the ball to (i.e., “left white”, “left orange”, “right white”, “right orange”, “left yellow”, “left pink”, “right yellow”, or “right pink”). If participants did not provide a response on a given trial, they were scored as making the worst decision (i.e., 1 out of 8). In both the 360VR and 2D video tasks, participants completed the ITC-SOPI and RSME at the conclusion of each sensory condition (s1, s2, and s3). Participants completed the 360VR and 2D video tasks in two separate sessions separated by ~ 12 days. This was due to participants’ work and/or study commitments making it difficult for participants to complete a single session of ~ 80 min before their usual ARF training. Not interrupting participants’ usual training was key to access skilled ARF players. The total time taken to complete both the 360VR and 2D video tasks, including the questionnaires, was approximately 80 min.

#### Dependent measures and statistical analysis

Decision-making performance, sense of presence, and cognitive load were the dependent variables in this study. The independent variables were task modality (360VR and 2D video), sensory conditions (s1, s2, and s3), contextual conditions within s2 (neutral and aggressive), auditory conditions within s3 (congruent and incongruent), and skill level (higher-skilled and lesser-skilled).

To address hypothesis 1, a Tobit regression was performed in STATA Statistical Software (Version 18, StataCorp, 2023). A Tobit regression was performed as it allows for the identification of relationships between censored dependent variables to a set of independent variables (Williams, 2016). Skill level (higher-skilled and lesser-skilled), modality (360VR and 2D video), and sensory condition (s1, s2, and s3) were included as fixed effects and individual participants were included as repeated factors. To address hypotheses 2 and 3, two separate Tobit regressions were performed with skill level (higher-skilled and lesser-skilled), modality (360VR and 2D video) and either context (s2, neutral and aggressive) or audio (s3, congruent and incongruent) included as fixed effects, and individual participants included as repeated factors. Significant main and interaction effects were investigated using pairwise comparisons with a Bonferroni correction and the alpha level was set at 0.05. Related to hypotheses 1 to 3, parametric one-sample t-tests or non-parametric Wilcoxon one-sample signed-rank tests were run to determine whether decision-making performance was significantly above, at, or below the 12.5% guessing level for an eight-choice task (see Supplementary Material B for this analysis). Normality checks were performed to determine whether skewness and kurtosis were ± 1.96 as per Field (2018) and therefore informed whether parametric or non-parametric tests were used. For these tests, the alpha level was set to 0.01 to guard against familywise error across the multiple one-sample comparisons (Field, 2018; Perneger, 1998). Cohen’s *d* or *r* was calculated for the one-sample t-tests or Wilcoxon one-sample signed-rank tests, respectively, to indicate the magnitude of response accuracy above chance (Field, 2018). For Cohen’s *d*, 0.20 indicates a small effect, 0.50 indicates a moderate effect, and 0.80 indicates a large effect. For Cohen’s *r*, 0.10, 0.30, and 0.50 signifies a small, moderate, and large effect, respectively (Cohen, 2013).

To address hypothesis 4, four separate Generalised Estimating Equations (GEE) were run in SPSS (IBM SPSS Statistics, Version 27, IBM Corp., New York United States of America) to examine differences in spatial presence, engagement, ecological validity, and negative effects. Modality (360VR and 2D video), sensory condition (s1, s2, and s3), and skill level (higher-skilled and lesser-skilled) were included as fixed effects, whilst individual participants were included as a repeated factor. To address hypothesis 5, differences in cognitive load were also examined using a GEE, with modality, sensory condition, and skill level included as fixed effects and individual participants as a repeated factor. As spatial presence, engagement, ecological validity, and negative effects, as well as cognitive load, are continuous measures, linear GEEs were performed. Residuals were checked to ensure they were within ± 3.29 for skewness and kurtosis (Ballinger, 2004; Field, 2018). GEEs were used as they facilitate the correct modelling of repeated observations and allow for non-normal distribution models (Ghisletta & Spini, 2004). For these analyses, any significant main and interaction effects were followed up using Bonferroni corrected pairwise comparisons, with a significance alpha level set at 0.05.

## Results

### Decision-making performance

In relation to hypothesis 1 (multi-sensory information), Tobit regression did not detect a significant three-way interaction between skill × modality × sensory condition on response accuracy, χ^2^(2) = 0.70, *p* = .703. Tobit regression also did not detect a main effect of skill [higher-skilled, *M* = 80.38%, *SE* = 0.86%; lesser-skilled, *M* = 79.00%, *SE* = 0.54%, χ^2^ (1) = 1.61, *p* = .205] and modality [360VR, *M* = 78.88%, *SE* = 0.54%; 2D video, *M* = 80.38%, *SE* = 0.55%, χ^2^ (1) = 0.90, *p* = .343] on response accuracy.

Tobit regression detected a significant main effect for sensory condition, χ^2^ (1) = 29.19, *p* < .001, with response accuracy significantly higher in vision only (*M* = 85.17%, *SE* = 0.59%) compared to both vision-contextual (*M* = 76.79%, *SE* = 0.69%, *p* < .001) and vision-contextual-auditory (*M* = 77.01%, *SE* = 0.70%, *p* < .001) conditions. There were no significant differences detected between vision-contextual and vision-contextual-auditory sensory conditions (*p* = 1.00).

**Fig. 2:**
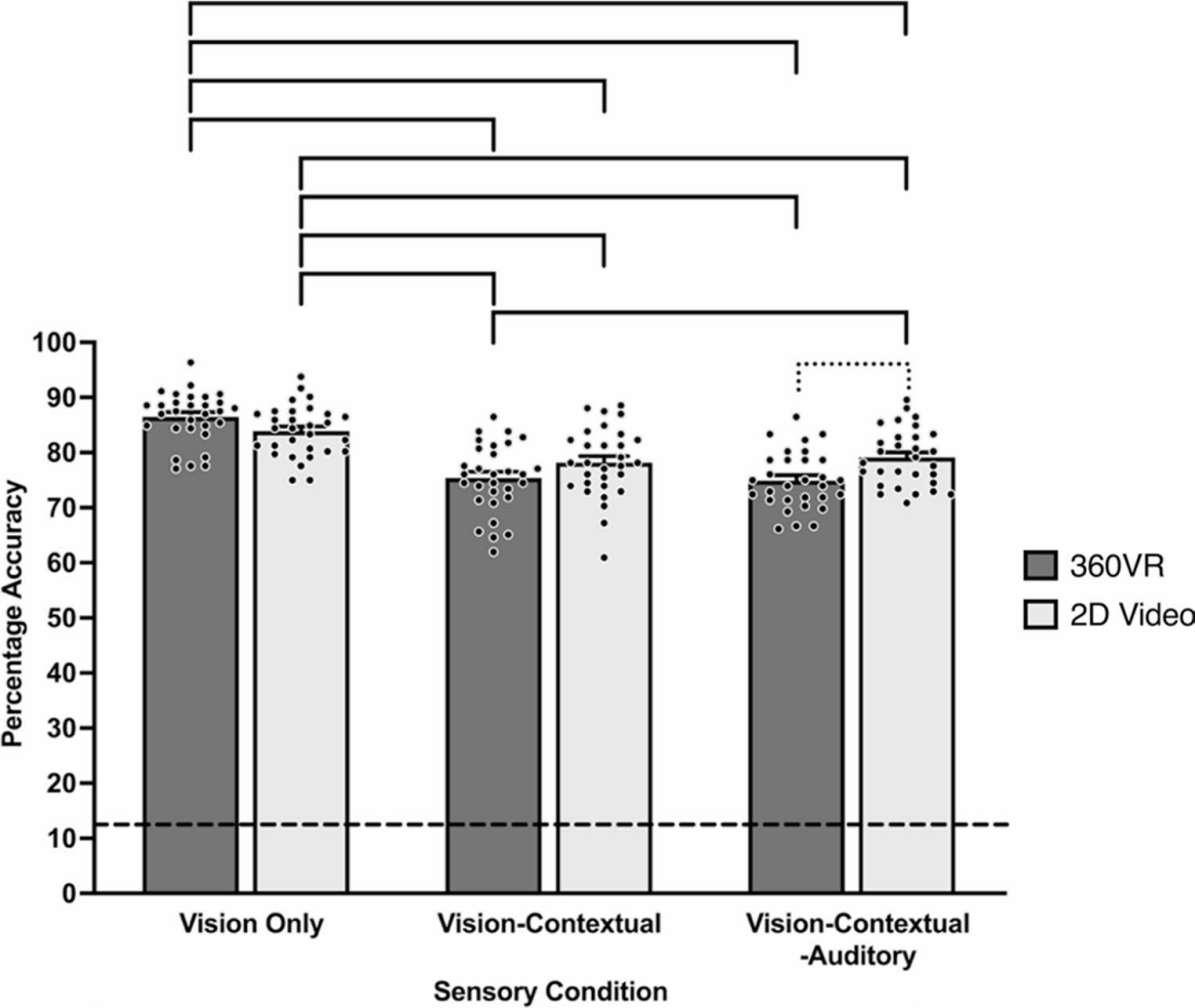
Mean decision-making response accuracy across sensory conditions and modality. Bracketed groupings indicate significant differences (*p* < .05). Dotted bracketed grouping highlights significant within sensory condition difference across modality (*p* < .05). Dashed line denotes the guessing level (12.5%). Error bars indicate standard error. Individual participant data points are represented by dots

Furthermore, no significant two-way skill × modality, χ^2^ (1) = 0.16, *p* = .688, or skill × sensory condition, χ^2^ (2) = 4.54, *p* = .103 interactions were detected. However, there was a significant two-way modality × sensory condition interaction on decision-making response accuracy, χ^2^ (2) = 6.35, *p* = .042 (see Fig. 2). Specifically, response accuracy in both 360VR vision only (*M* = 86.46%, *SE* = 0.78%) and 2D video vision only (*M* = 83.89%, *SE* = 0.88%) was significantly higher than vision-contextual in 360VR (*M* = 75.38%, *SE* = 0.99%) and 2D video (*M* = 78.19%, *SE* = 0.95%), as well as vision-contextual-audio in 360VR (*M* = 74.93%, *SE* = 1.03%) and 2D video (*M* = 79.10%, *SE* = 0.94%, *p*s < 0.05). Further, response accuracy was significantly greater in 2D video vision-contextual-auditory compared to 360VR vision-contextual (*p* = .039). Finally, response accuracy in vision-contextual-auditory was significantly higher in 2D video compared to 360VR *(p* = .033).

In relation to hypothesis 2 (contextual information), no significant three-way interaction of skill × context condition (s2) × modality on response accuracy, χ^2^ (1) = 0.25, *p* = .614 was detected. Further, the Tobit regression did not detect a significant main effect of skill [higher-skilled, *M* = 78.50%, *SE* = 0.94%; lesser-skilled, *M* = 75.13%, *SE* = 0.99%, χ^2^ (1) = 2.25, *p* = .134] or modality [360VR, *M* = 75.50%, *SE* = 0.99%; 2D video, *M* = 78.25%, *SE* = 0.95%, χ^2^ (1) = 2.69, *p* = .101] on response accuracy.

Tobit regression detected a significant main effect of context condition, χ^2^ (1) = 10.92, *p* = .001, with response accuracy significantly higher in neutral (*M* = 83.25%, *SE* = 0.90%) compared to aggressive (*M* = 70.38%, *SE* = 0.98%) context.

**Fig. 3:**
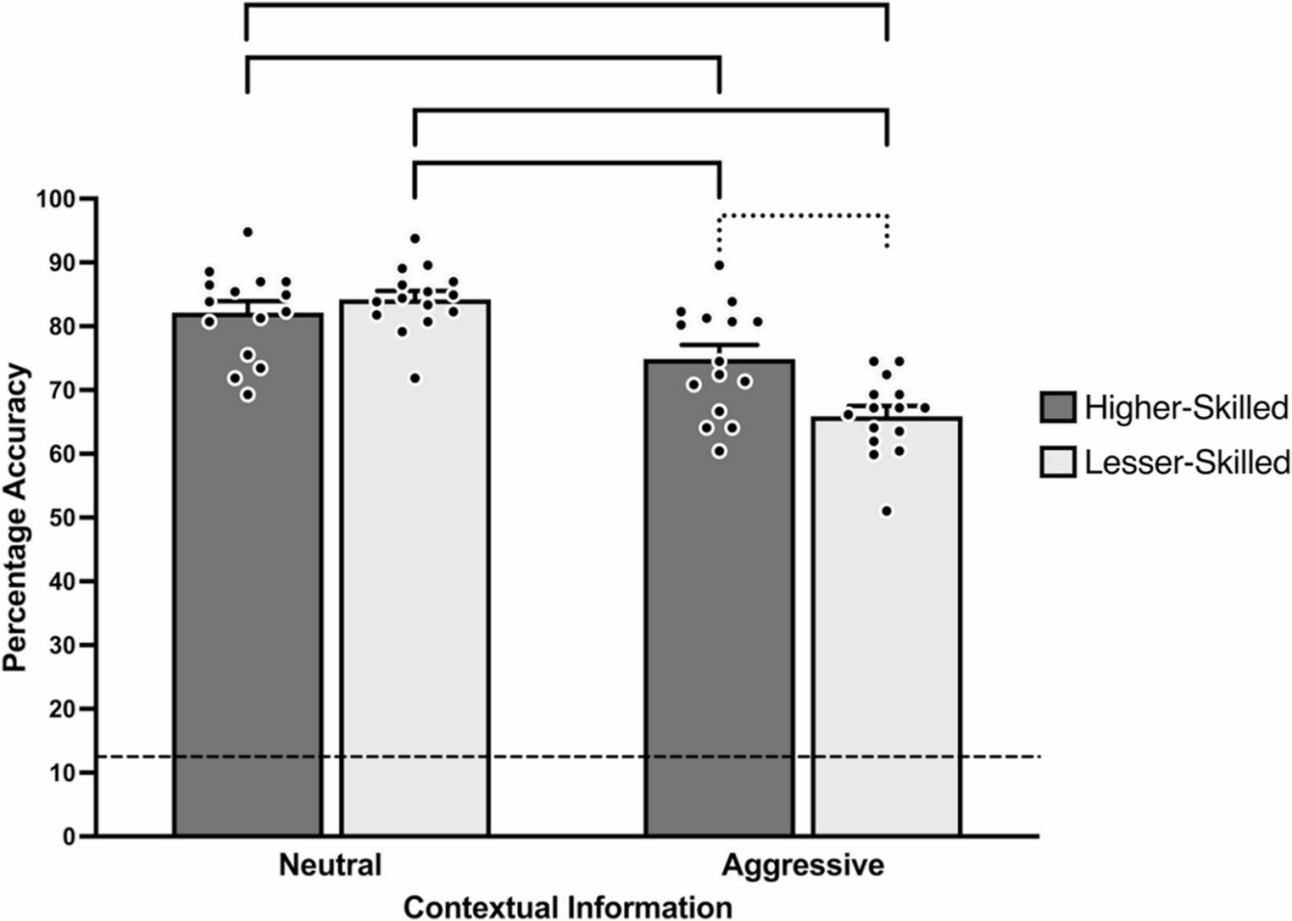
Mean decision-making response accuracy across skill and contextual information in sensory condition two. Bracketed groupings indicate significant difference (*p* < .05). Dotted bracketed grouping highlights significant within context condition difference across skill level (*p* < .05). Dashed line denotes the guessing level (12.5%). Error bars indicate standard error. Individual participant data points are represented by dots

There was a significant two-way skill × context condition interaction, χ^2^ (1) = 8.80, *p* = .003 detected. Specifically, response accuracy was significantly greater for higher-skilled neutral (*M* = 82.13%, *SE* = 1.33%) compared to higher-skilled aggressive (*M* = 74.88%, *SE* = 1.26%) and lesser-skilled aggressive (*M* = 65.88%, *SE* = 1.40%, *p*s < .001, see Fig. 3). Furthermore, response accuracy was significantly higher for lesser-skilled neutral (*M* = 84.25%, *SE* = 1.21%) compared to both higher-skilled aggressive and lesser-skilled aggressive (*p*s < 0.001, see Fig. 3). Finally, response accuracy was significantly greater in higher-skilled aggressive compared to lesser-skilled aggressive (*p* < .001).

There was no significant skill × modality two-way interaction, χ^2^ (1) = 1.29, *p* = .256, nor a significant modality × context condition two-way interaction, χ^2^ (1) = 0.00, *p* = .950 detected.

**Fig. 4:**
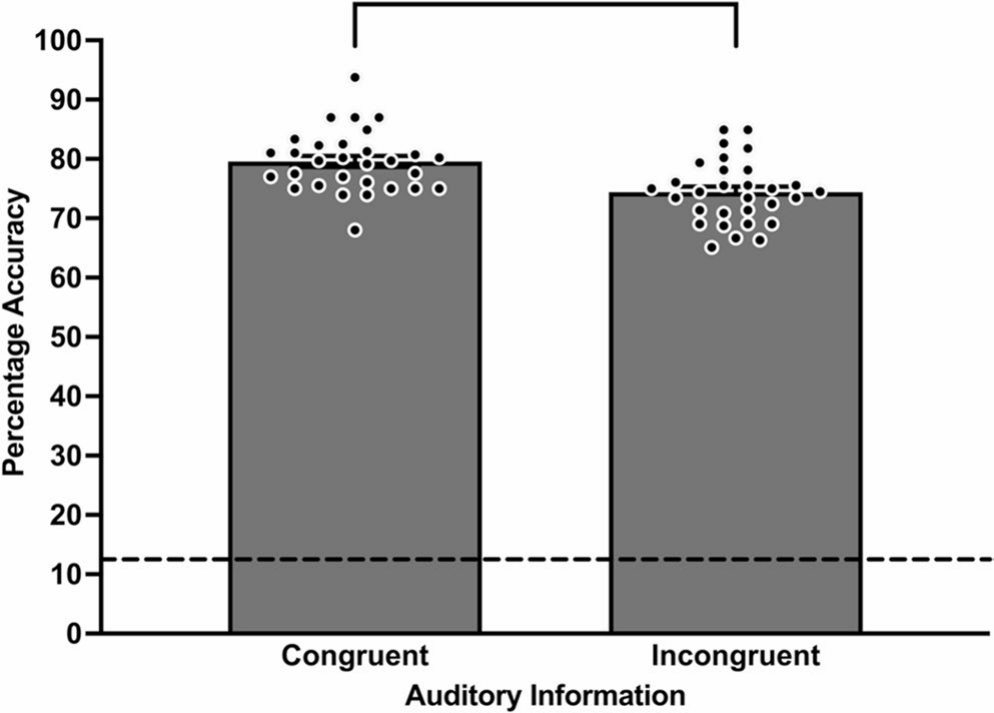
Mean decision-making response accuracy across auditory information in sensory condition three. Bracketed groupings indicate significant differences between auditory conditions (*p* < .05). Dashed line denotes the guessing level (12.5%). Error bars indicate standard error. Individual participant data points are represented by dots

In relation to hypothesis 3 (auditory information), Tobit regression did not detect a significant three-way interaction of skill × modality × auditory information (s3) on decision-making response accuracy, χ^2^ (1) = 0.04, *p* = .834. However, there was a significant main effect of audio on response accuracy, with significantly higher performance in congruent audio (*M* = 79.50%, *SE* = 0.93%) compared to incongruent audio (*M* = 74.38%, *SE* = 1.04%), χ^2^ (1) = 5.60, *p* = .018 (see Fig. 4).

Tobit regression did not detect a significant effect for skill [higher-skilled, *M* = 78.38%, *SE* = 0.98%; lesser-skilled, *M* = 75.63%, *SE* = 0.99%, χ^2^ (1) = 0.95, *p* = .329] nor modality [360VR, *M* = 74.88%, *SE* = 1.03%; 2D video, *M* = 79.13%, *SE* = 0.94%, χ^2^ (1) = 0.81, *p* = .367] on response accuracy when participants were presented with congruent and incongruent auditory information. Furthermore, no significant two-way interactions between skill × modality, χ^2^ (1) = 0.08, *p* = .782, skill × auditory information, χ^2^ (1) = 0.00, *p* = .952, nor modality × auditory information, χ^2^ (1) = 0.51, *p* = .474 were detected.

### Sense of presence

#### Spatial presence

**Fig. 5:**
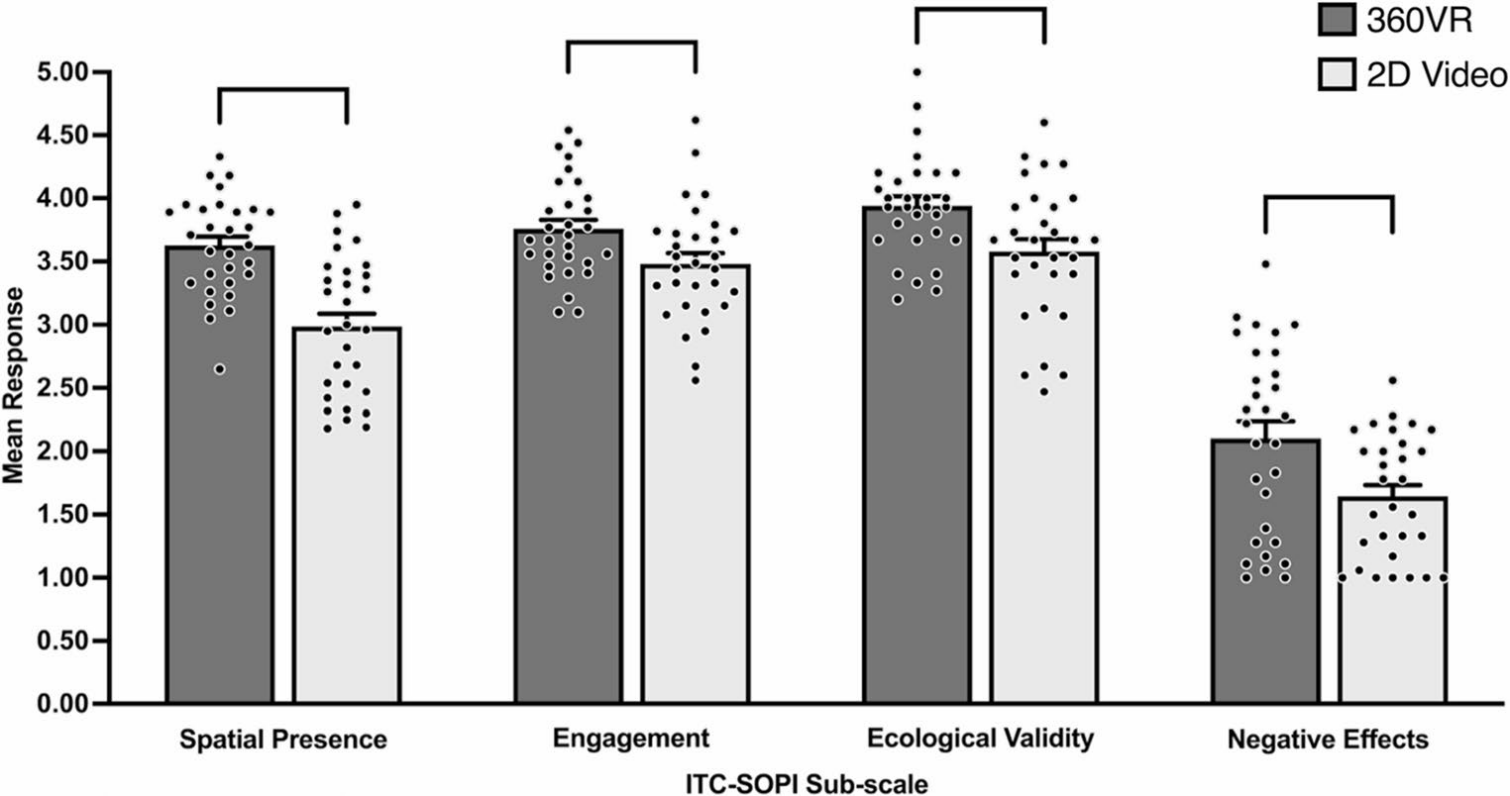
Sense of presence across task modality. Bracketed groupings indicate significant difference (*p* < .05). Error bars indicate standard error. Individual participant data points are represented by dots

In relation to hypothesis 4, the GEE for spatial presence did not detect a significant three-way interaction between skill × sensory condition × modality, χ^2^ (2) = 2.06, *p* = .357. Furthermore, the main effects of skill [higher-skilled, *M* = 3.25, *SE* = 0.10; lesser-skilled, *M* = 3.36, *SE* = 0.11, ^χ2^ (1) = 0.55, *p* = .460] and sensory condition [vision only, *M* = 3.25, *SE* = 0.08; vision-contextual, *M* = 3.29, *SE* = 0.08; vision-contextual-auditory, *M* = 3.38, *SE* = 0.07, χ^2^ (2) = 5.79, *p* = .055] did not detect a significant effect on spatial presence. However, there was a significant main effect of modality, with spatial presence reported significantly higher in 360VR (*M* = 3.63, *SE* = 0.07) compared to 2D video (*M* = 2.99, *SE* = 0.10), χ^2^ (1) = 56.08, *p* < .001 (see Fig. 5). The GEE analysis did not detect a significant two-way interaction for skill × sensory condition, χ^2^ (2) = 4.67, *p* = .097, skill × modality, χ^2^ (1) = 1.29, *p* = .257, or sensory condition × modality on spatial presence, χ^2^ (2) = 1.94, *p* = .380.

#### Engagement

The GEE did not detect a significant three-way interaction between skill × sensory condition × modality on engagement χ^2^ (2) = 2.85, *p* = .241. There was also no significant skill [higher-skilled, *M* = 3.61, *SE* = 0.11; lesser-skilled, *M* = 3.63, *SE* = 0.09, χ^2^ (1) = 0.02, *p* = .885] or sensory condition [vision only, *M* = 3.61, *SE* = 0.08; vision-contextual, *M* = 3.62, *SE* = 0.08; vision-contextual-auditory, *M* = 3.63, *SE* = 0.07, χ^2^ (2) = 0.24, *p* = .887] main effects detected. However, the GEE did indicate a significant main effect of modality, with engagement reported significantly higher in 360VR (*M* = 3.76, *SE* = 0.07) compared to 2D video (*M* = 3.48, *SE* = 0.08), χ^2^ (1) = 26.54, *p* < .001 (see Fig. 5). Further, the GEE did not detect a significant two-way interaction for skill × modality, χ^2^ (1) = 3.54, *p* = .060, or sensory condition × modality on engagement, χ^2^ (2) = 1.76, *p* = .414, but there was a significant skill × sensory condition interaction, χ^2^ (2) = 8.71, *p* = .013. However, this effect was no longer significant with post hoc comparisons using a Bonferroni correction.

#### Ecological validity

The GEE did not detect a significant three-way skill × sensory condition × modality interaction on ecological validity, χ^2^ (2) = 3.95, *p* = .139. There was a significant main effect of modality, χ^2^ (1) = 21.57, *p* < .001, with significantly higher ecological validity reported in 360VR (*M* = 3.94, *SE* = 0.07) compared to 2D video (*M* = 3.58, *SE* = 0.10, see Fig. 5). However, the main effect of skill [higher-skilled, *M* = 3.69, *SE* = 0.11; lesser-skilled, *M* = 3.83, *SE* = 0.11, χ^2^ (1) = 0.91, *p* = .339], and sensory condition [vision only, *M* = 3.81, *SE* = 0.09; vision-contextual, *M* = 3.76, *SE* = 0.07; vision-contextual-auditory, *M* = 3.71, *SE* = 0.08, χ^2^ (2) = 2.01, *p* = .367] did not detect a significant effect on ecological validity.

The GEE indicated a significant two-way interaction between modality × sensory condition, χ^2^ (2) = 6.18, *p* = .045 (see Supplementary Material C). Post-hoc tests revealed ecological validity was rated significantly higher in 360VR vision only (*M* = 3.74, *SE* = 0.08) compared to 2D video vision-contextual (*M* = 3.49, *SE* = 0.08, *p* = .000) and 2D video vision-contextual-auditory (*M* = 3.46, *SE* = 0.10, *p* = .004). In addition, ecological validity was significantly higher in 360VR vision-contextual (*M* = 3.74, *SE* = 0.08) compared to 2D video vision only (*M* = 3.49, *SE* = 0.09, *p* = .004), 2D video vision-contextual (*p* < .001), and 2D video vision-contextual-auditory (*p* = .004). Further, ecological validity was rated significantly higher in 360VR vision-contextual-auditory (*M* = 3.80, *SE* = 0.06) compared to both 2D video vision-contextual and 2D video vision-contextual-auditory (*p*s < .001). The two-way interactions of skill × modality, χ^2^ (1) = 0.29, *p* = .588, and skill × sensory condition, χ^2^ (2) = 5.86, *p* = .053, did not detect a significant effect on ecological validity.

#### Negative effects

The GEE did not detect a significant three-way skill × sensory condition × modality interaction on negative effects, χ^2^ (2) = 0.68, *p* = .714. Further, no significant main effect of skill [higher-skilled, *M* = 1.85, *SE* = 0.14; lesser-skilled, *M* = 1.89, *SE* = 0.15, χ^2^ (1) = 0.04, *p* = .884] or sensory condition [vision only, *M* = 1.93, *SE* = 0.11; vision-contextual, *M* = 1.86, *SE* = 0.10; vision-contextual-auditory, *M* = 1.83, *SE* = 0.10, χ^2^ (2) = 2.80, *p* = .246] on negative effects was detected. However, there was a significant main effect of modality, with negative effects being significantly higher in 360VR (*M* = 2.10, *SE* = 0.13) compared to 2D video (*M* = 1.64, *SE* = 0.09), χ^2^ (1) = 22.01, *p* < .001 (see Fig. 5). Finally, GEE did not detect significant two-way interactions of skill × modality, χ^2^ (1) = 1.84, *p* = .175, skill × sensory condition χ^2^ (2) = 2.01, *p* = .349, or modality × sensory condition on negative effects, χ^2^ (2) = 0.68, *p* = .712.

### Cognitive load

In relation to hypothesis 5, GEE did not detect a significant three-way interaction of skill × sensory condition × modality on cognitive load, χ^2^ (2) = 1.98, *p* = .372. However, GEE detected a significant main effect of modality, χ^2^ (1) = 4.32, *p* = .038, with cognitive load rated significantly higher in 360VR (*M* = 73.77, *SE* = 4.06) compared to 2D video (*M* = 68.17, *SE* = 4.45). In addition, GEE detected a significant main effect of sensory condition on cognitive load, χ^2^ (2) = 19.15, *p* < .001. Participants rated cognitive load significantly lower in vision only (*M* = 66.32, *SE* = 3.99) compared to vision-contextual (*M* = 70.95, *SE* = 4.09) and vision-contextual-auditory (*M* = 75.63, *SE* = 4.50), as well as significantly lower in vision-contextual compared to vision-contextual-auditory, *p*s < .05. Moreover, GEE did not detect a significant main effect of skill on cognitive load [higher-skilled, *M* = 68.66, *SE* = 5.71; lesser-skilled, *M* = 73.28, *SE* = 5.73, χ^2^ (1) = 0.33, *p* = .568]. In addition, the GEE did not detect a significant two-way interaction between skill × sensory condition, χ^2^ (2) = 3.65, *p* = .162, or modality × sensory condition, χ^2^ (2) = 2.90, *p* = .235. However, there was a significant two-way interaction between skill × modality, χ^2^ (1) = 4.50, *p* = .045, with higher-skilled participants reporting significantly greater cognitive load in 360VR (*M* = 74.31, *SE* = 5.68) compared to 2D video (*M* = 63.01, *SE* = 6.46, *p* = .045), but not lesser-skilled (360VR, *M* = 73.22, *SE* = 5.80, 2D video, *M* = 73.33, *SE* = 6.12, *p* = 1.00).

## Discussion

The purpose of this study was to investigate whether increased sensory information and immersion influences decision-making performance, presence, and cognitive load across different levels of expertise. To achieve this aim, higher- and lesser-skilled ARF players completed a sport-specific decision-making task, which presented visual information only, visual and contextual information, and visual, contextual and auditory information in both 360VR and 2D video. The findings of this study provide valuable insights for the design and implementation of simulated environments to assess, and potentially enhance, decision-making skill in high-stakes domains.

### Decision-making performance

Hypothesis one predicted expertise would influence multi-sensory information use for decision-making, with superior performance under 360VR compared to 2D video. However, results did not find evidence that expertise influenced decision-making accuracy across sensory conditions (vision only; visual and contextual; visual, contextual and auditory). This finding contrasts the broader expertise literature, which indicates higher-skilled participants have a superior capability to integrate non-visual sources of information with visual cues to guide anticipation and decision-making (Runswick et al. 2018; Williams and Jackson 2019a). A potential reason for a lack of expertise effect could be due to the higher- and lesser-skilled participants in this study being closer on the skill continuum than previous research investigating perceptual-cognitive skill in sport, which usually compares higher-skilled athletes with novices (Glazier, 2017). Indeed, there is individual differences based evidence that some lesser-skilled athletes can outperform their higher-skilled counterparts on decision-making (e.g., Morris-Binelli et al., 2025) and anticipation tasks (e.g., Morris-Binelli et al., 2021). Our simulator modality prediction was also not supported as the results did not find differences in participants’ decision-making accuracy in 360VR compared to 2D video across all sensory conditions, with 2D video performance higher than 360VR when visual, contextual and auditory information was presented simultaneously. This finding extends upon previous studies reporting similar decision-making performance in visual-only tasks in 360VR and 2D video for elite level ARF umpires (Kittel et al., 2019) and skilled basketball players (Richard et al., 2022). That is, increased immersion provided by 360VR does not appear to influence decision-making accuracy in a bi-sensory (s2, vision-contextual) and multi-sensory (s3, vision-contextual-auditory) sport-specific task. As a key argument for the benefit of virtual reality (VR), such as 360VR, over 2D video is that increased immersion will influence performance of perceptual-cognitive skills (Düking et al., 2018; Harris et al., 2020; Kittel et al., 2024), our findings suggest immersion is not as critical for enhancing performance. Rather, what is likely more vital for decision-making is the provision of domain-specific perceptual information (Kalén et al., 2021; Müller et al., 2023), which was maintained across our 360VR and 2D video tasks.

Despite the lack of skill-based differences, decision-making performance significantly decreased as sensory information was added across 360VR and 2D video. Specifically, decision-making accuracy of lesser- and higher-skilled athletes was significantly higher in the visual-only compared to visual and contextual, as well as visual, contextual and auditory conditions. As this is the first study to our knowledge to simultaneously compare decision-making across uni-sensory, bi-sensory, and multi-sensory information sources, direct comparison of our findings to previous research is difficult. However, the decrease in decision-making performance from vision only to visual and contextual information differs from previous studies that have reported the provision of congruent contextual information to visual cues enhances anticipation performance in cricket batting (McRobert et al., 2011; Runswick et al., 2018), soccer field play (Gredin et al., 2021), and intercepting opponents’ attacking movements in karate (Milazzo et al., 2016). Alternatively, the lack of performance improvements from vision only to visual and contextual information in the current study aligns with Magnaguagno et al. (2022), who reported provision of explicit contextual information, in the form of opponent action preferences, did not influence decision-making accuracy in a European handball task. Therefore, our results build upon these findings and indicate the provision of contextual information may not always enhance decision-making accuracy. The rationale for our findings and those from Magnaguagno et al. (2022) suggests that contextual information only improves decision-making when it provides reliable predictive cues. When additional information is needed to be integrated with visual information, the processing demands may increase and attention to the most task-specific information may be reduced.

Interestingly, we found no significant differences in decision-making between vision-contextual (s2, bi-sensory) and vision-contextual-auditory (s3, multi-sensory) conditions. Previous studies have found experienced tennis players adjust their predictions of ball trajectory based upon the loudness of their opponent’s racquet-ball contact (Cañal-Bruland et al., 2018), whilst simulated grunting sounds from an opponent significantly reduce the accuracy of tennis players’ shot location predictions (Sinnett & Kingstone, 2010). In contrast, Allerdissen et al. (2017) found no differences in expert fencers’ prediction of opponent attack movements when provided with visual-only information or audio-visual information. Therefore, our findings extend upon Allerdissen et al. (2017) as they suggest the provision of auditory information, in the form of a teammate verbally directing the performer of where to pass the ball, provides limited information to guide decision-making, beyond that which is provided by visual and contextual cues. Alternatively, performers in our study may have considered the auditory information less relevant than visual and contextual information, and therefore, allocated it less attentional resources (Allerdissen et al., 2017). Collectively, our findings suggest inclusion of multi-sensory information, which is more representative to the competition environment, increased the amount of perceptual information that had to be processed and integrated by performers. This may have caused conflict or uncertainty in information use, which resulted in less accurate decisions compared to when only visual information was presented. This interpretation aligns with the proposition that the capability to integrate and weight task-relevant visual and non-visual information is a crucial component of perceptual-cognitive skill, not the mere presence of additional non-visual information (Gredin et al. 2023; Morris-Binelli and Müller 2017; Williams and Jackson 2019b).

Hypothesis two predicted expertise would influence visual and contextual information use with superior performance under 360VR compared to 2D video. To address this hypothesis, decision-making performance was compared when only visual and contextual information (s2) was presented in 360VR and 2D video. Findings from this analysis supported our hypothesis as higher-skilled players made significantly more accurate decisions than lesser-skilled players when presented with aggressive contextual information. The contextual information manipulation in our task was designed to provide game-specific information (score and time) that required participants to adjust their decision-making. That is, from a more conservative decision-making strategy to maintain possession of the ball and protect their team’s lead over the opposition (neutral), to a strategy that required riskier aggressive decisions and movement of the ball towards central locations of the field to increase their team’s capability to score quickly and win the game. Recent studies using an immersive CAVE have demonstrated expert European handball players are superior to lesser-skilled players in their capability to detect and utilise contextual information (strong or weak defender) to guide accurate decision-making, without being explicitly told that such contextual information is present within the task (Magnaguagno & Hossner, 2020; Magnaguagno et al., 2022). As participants in our study were not explicitly told to adjust their decision-making strategy, our findings align with the proposition that the superior capability to use self-generated knowledge informed by contextual information to guide decision-making is an important aspect of perceptual-cognitive expertise (Magnaguagno & Hossner, 2020; Magnaguagno et al., 2022). A unique finding of the current study is the lack of differences in the use of contextual information across simulator modalities for either skill group, highlighting that the perception and use of non-visual information is similar across higher- and lower-immersive modalities. Accordingly, our findings provide evidence that the superior capability of higher-skilled players to utilise contextual information, in the form of game score and time, appears independent to immersion. It could be, however, that use of other XR technology such as CAVE, which provide performers with the opportunity to make decisions with a sport-specific motor response (e.g., kick), may lead to performance differences across simulators with similar levels of immersion, such as 360VR. Nonetheless, a focus of the current study was to investigate whether higher immersion (360VR) compared to lower immersion (2D video) influences decision-making performance, rather than a comparison of varying degrees of perception-action coupled responses on decision-making.

Hypothesis three predicted expertise would influence auditory information use, with superior performance under 360VR compared to 2D video. The findings did not support this hypothesis as no expertise differences were detected in decision-making performance when participants were presented with congruent and incongruent auditory information across 360VR and 2D video. This finding contrasts the skill-based differences reported in experiment 2 of Klatt and Smeeton (2020), where expert volleyball players outperformed amateurs when presented with visual-auditory congruent and incongruent information. A potential reason for our lack of skill-based differences could be due to our lesser- and higher-skilled participants both competing at a semi-professional level, and therefore, being closer together on the skill continuum than the participants in Klatt and Smeeton (2020). Additionally, each trial in our study presented continuous visual, contextual, and auditory information over an approximately 6 to 8 s duration, as typically occurs in a match. In contrast, each trial in Klatt and Smeeton (2020) presented still images for either 50 ms, 200 ms, or 350 ms with a single auditory cue. Consequently, the longer stimulus presentation in our study may have decreased any expertise differences as both higher- and lesser-skilled participants had more opportunity to update their decision-making based upon the unfolding perceptual information. When skill level and simulator modality were collapsed, we did find that decision-making accuracy was significantly higher when the auditory information presented was congruent with the available vision-contextual information, compared to when it was incongruent, which aligns with experiment 1 findings in Klatt and Smeeton (2020). Therefore, irrespective of the lack of an expertise effect, our findings suggest that the capability to effectively filter erroneous auditory information is important for superior decision-making. Crucially, this capability does not appear to be influenced by higher (360VR) and lower (2D video) degrees of simulator immersion.

### Sense of presence

Hypothesis four predicted increased sensory information (vision only to vision-contextual to vision-contextual-auditory) would result in greater sense of presence in 360VR compared to 2D video, irrespective of expertise. As expected, spatial presence, engagement, and ecological validity were rated significantly higher in 360VR compared to 2D video, regardless of skill. This aligns with the broader XR literature, whereby greater immersion facilitated by HMDs leads to heightened levels of various dimensions of presence (Harris et al., 2020; Kittel et al., 2019; Loiseau Taupin et al., 2023; Ochs & Sonderegger, 2022). In contrast to our hypothesis, provision of multi-sensory information did not increase spatial presence or engagement in either 360VR or 2D video; hence our hypothesis was rejected. Interestingly, ecological validity was rated significantly higher in 360VR vision-contextual compared to 2D video vision-contextual, whilst 360VR vision-contextual-auditory was rated significantly higher than 2D video vision-contextual-auditory. As this is the first study in sport to our knowledge to compare sense of presence across uni-sensory to multi-sensory perceptual information in 360VR and 2D video, these findings provide unique evidence to suggest the provision of contextual and auditory information, which is more representative of the in-situ setting, does not enhance spatial presence, engagement, and ecological validity in 360VR beyond the provision of domain-specific visual information only. These findings contrast evidence from studies implementing a vehicle wheel change (Cooper et al., 2018) and memory (Dinh et al., 1999) task in virtual environments, whereby an increase in multi-sensory information resulted in greater sense of presence. Our contrasting findings could be due to vision being the dominant sensory system used for fast-paced decision-making in sport (Williams and Jackson 2019a), therefore, resulting in additional contextual and auditory information having little influence on sense of presence. The current study also found that negative effects were rated significantly higher in 360VR compared to 2D video, irrespective of sensory condition or skill. It is important to note, however, that despite negative effects being rated significantly higher in 360VR compared to 2D video, ratings in 360VR were relatively low and similar to levels reported in Vawser et al. (2025).

Collectively, these findings provide an important theoretical implication. Whilst increased immersion provided by 360VR leads to greater sense of presence compared to 2D video, this does not appear to influence decision-making performance, even with the provision of representative multi-sensory perceptual information. In fact, although ecological validity was rated as significantly higher in 360VR compared to 2D video vision-contextual-auditory (s3), decision-making performance was significantly more accurate in the latter modality. This contrasts the proposition that increased immersion in XR necessarily provides a more effective technology for investigating perceptual-cognitive skills such as decision-making (Carruth, 2017; Drew, 2021), at least within the constraints of the present task design.

### Cognitive load

The final hypothesis predicted that cognitive load would be higher for lesser-skilled players compared to higher-skilled players across multi-sensory information, with increased load in 360VR compared to 2D video. The findings, however, did not support this hypothesis. When skill level was collapsed, cognitive load significantly increased as multi-sensory information was added, and this was not influenced by simulator modality. This finding suggests adding multi-sensory information, and therefore, increasing the extraneous load of the task, plays an important role in increasing cognitive load as per Cognitive Load Theory (Paas et al., 2003). Interestingly, higher-skilled participants reported greater cognitive load in 360VR compared to 2D video when multi-sensory information was collapsed, with no difference for lesser-skilled participants. This expertise effect, coupled with the finding that cognitive load was significantly higher in 360VR than 2D video when skill and sensory modality were collapsed, suggest that as tasks become more representative through the simulation of multi-sensory information, immersive properties of 360VR begin to influence cognitive load. This may explain the lack of differences in cognitive load in previous sport tasks, which only presented visual information. That is, Hoyne et al. (2026) reported no significant differences in cognitive load in an ARF task presented in 360VR and 2D video, whilst Loiseau Taupin et al. (2023) found no significant differences in cognitive load in a boxing task displayed in 360VR and 2D video. These findings suggest that as simulators, particularly VR, become more representative, this will likely influence performers’ cognitive load, and higher-skilled performers’ cognitive load may increase more so than lesser-skilled performers.

### Theoretical, technological, and practical implications

There are several theoretical, technological, and practical implications from this study. First, the lack of influence that immersion and presence had on multi-sensory decision-making performance indicates that a highly immersive simulated environment is not required to assess, and potentially enhance, decision-making skill. Importantly, increasing the representativeness of the simulated environment through the inclusion of multi-sensory information did not tease out any potential effect that enhanced immersion and presence may have on decision-making performance in an invasion sport-specific task. Accordingly, if financial resources restrict an organisation’s capability to acquire a 360-degree camera and HMDs to create and view an immersive display, lower cost standard video cameras and projectors still provide a suitable option to assess decision-making skill. Second, as the findings indicated that 360VR is perceived by users as more engaging and ecologically valid than 2D video, despite both presenting the same representative perceptual information, a key benefit of 360VR is that it may increase athlete acceptance to use simulated environments to assess and enhance decision-making skill off the field (Kittel et al., 2026; Mascret et al., 2022). In terms of technology implementation in sport and other high-performance domains (e.g., defence, law enforcement, medicine), this is an important consideration as users’ perceptions of technology can influence the likelihood of adoption and continued use (Mascret et al., 2022). Third, regardless of the degree of immersion and presence provided by a simulated environment (e.g., 360VR or 2D video), the inclusion of representative multi-sensory perceptual information (e.g., visual, contextual, auditory) is vital to assess performers’ decision-making capabilities (Gredin et al., 2023; Morris-Binelli & Müller, 2017). This is important to ensure the simulated environment has a high degree of psychological fidelity, which is a key mechanism of perceptual-cognitive expertise (Harris et al., 2020; Müller et al., 2023). Further, as differences in decision-making performance between performers who were closer together on the skill continuum became apparent when different sources of perceptual information were added (i.e., visual and contextual), tasks which only include one source of perceptual information may not have the sensitivity to detect skill-based differences in performance. Therefore, failure to model multi-sensory information, and thereby ensure adequate psychological fidelity, may result in simulated environments that do not capture real-world performance and have limited capability to enhance decision-making skill (Müller et al., 2023). Finally, as the inclusion of multi-sensory information in simulated environments increased participants’ cognitive load, the design and implementation of such assessment and training programs to enhance decision-making skill need to consider performers’ cognitive load as part of workload management to guide the programming of training and recovery (Champion et al., 2023).

### Future research, limitations, and conclusions

A potential limitation of this study is that it did not include other types of contextual (e.g., opponent action tendencies) and auditory (e.g., physical exertion sounds by an opponent about to tackle a player) information, which may interact with immersion to influence decision-making performance in a different manner to that observed here. Nonetheless, this was the first study to our knowledge to investigate the impact of multi-sensory information use on decision-making performance, presence, and cognitive load across differing degrees of immersive simulators, and we chose to model common sources of contextual and auditory information present in game. Additionally, the decision-making task used in this study were all staged scenarios, which although allowed for greater experimental control, these scenarios may not entirely represent the dynamic nature of decision-making in invasive sports. Furthermore, the task focused upon mark-disposal decision-making on one location of an ARF field (i.e., middle-side). It is possible that multi-sensory information may be utilised differently in other locations (e.g., forward half) and contexts within a game (e.g., centre bounce contests) and immersion may have a different influence on performance to that observed in the present study. In addition, two of the participants who were also actors in the filmed scenarios may have recognised running patterns in the task, which could have influenced their decisions. However, during filming, which was approximately one year prior to this study, these participants were unaware of the outcomes of the running patterns. Accordingly, this coupled with the time between filming and completion of the decision-making tasks would limit the likelihood that they would have remembered the running patterns and be aware of the associated scoring. Importantly, a requirement from the ARF club was that those involved in the development of the research were also invited to participate in the study. A further limitation is that response time was not measured, as decision-making performance was assessed based on response accuracy; future research may benefit from examining both the speed and accuracy of responses to better understand how athletes evaluate and integrate multi-sensory perceptual information during decision-making across highly immersive and less immersive simulators. Finally, it is important to acknowledge that a limitation of the simulators may be their lack of physical representativeness as neither enable action-based responses. However, evidence exists that overt perception-action coupling is not a crucial determinant of decision-making skill (Ranganathan & Carlton, 2007; Vignais et al., 2015). Furthermore, verbal responses are sufficient to discriminate expert performance and activate motor cortical regions (Aglioti et al., 2008; Kalén et al., 2021).

Future research should investigate whether training decision-making in highly immersive compared to less immersive simulated environments, which include representative multi-sensory information, leads to different learning and transfer benefits. Indeed, there is evidence that perceptual-cognitive skills, such as decision-making, can be enhanced through the use of 2D video simulators, with positive transfer to the field (see Müller et al., 2024). However, there is a dearth of research investigating whether VR environments, such as 360VR, offer any additional benefit to decision-making skill learning and transfer than 2D video. The few sport-specific decision-making VR studies have provided mixed results, with one indicating VR leads to greater learning benefit than 2D video (Fortes et al., 2021), whilst two others indicate VR provides limited learning benefit over 2D video (Kittel et al., 2020; Pagé et al., 2019). Importantly, these studies included visual-only information in the test and training stimulus. Therefore, understanding how multi-sensory information, immersion, and presence influence decision-making skill enhancement and transfer to field-based tasks will provide important theoretical and practical insights regarding the design and implementation of simulation-based training programs to maximise decision-making skill in time-constrained domains.

XR simulators are becoming an increasingly popular technology to assess and enhance perceptual-cognitive skill in sport and other domains (e.g., law enforcement). This is largely due to its capability to present users with engaging and exciting performance scenarios that are perceived to better replicate the real world than traditional 2D video-based simulators. The findings from the current study, however, indicate that the proposed technological benefits of XR, that is, greater immersion and presence, have little influence on decision-making skill in a time-constrained task. Rather, what appears to be key for decision-making skill is the capability to pick-up domain-specific multi-sensory information, and this can occur at similar degrees in highly immersive and less immersive modalities. Accordingly, the design of simulated environments, whether 360VR or 2D video, must prioritise the inclusion of representative perceptual information to maintain psychological fidelity. This will ensure simulators are designed to target a key mechanism of expertise. At present, the differentiating benefit of 360VR over 2D video appears to be the enhanced capability of the former technology to engage users, and potentially, increase the likelihood that performers will want to assess and train their decision-making capabilities off the field. A crucial next step is to systematically explore whether 360VR, designed to maintain sound psychological fidelity, provides benefit to accelerate perceptual-cognitive-motor skill.

## Data Availability

The data that support the findings of this study are available upon reasonable request from the corresponding author. The data are not publicly available as it contains information that could compromise the privacy of research participants.
